# A short G1 phase imposes constitutive replication stress and fork remodelling in mouse embryonic stem cells

**DOI:** 10.1038/ncomms10660

**Published:** 2016-02-15

**Authors:** Akshay K. Ahuja, Karolina Jodkowska, Federico Teloni, Anna H. Bizard, Ralph Zellweger, Raquel Herrador, Sagrario Ortega, Ian D. Hickson, Matthias Altmeyer, Juan Mendez, Massimo Lopes

**Affiliations:** 1 Institute of Molecular Cancer Research, University of Zurich, Zurich CH-8057, Switzerland; 2 DNA Replication Group, Molecular Oncology Programme, CNIO, Madrid E-28029, Spain; 3 Institute of Veterinary Biochemistry and Molecular Biology, University of Zurich, Zurich CH-8057, Switzerland; 4 Department of Cellular and Molecular Medicine, Center for Chromosome Stability and Center for Healthy Aging, University of Copenhagen, Panum Institute, Copenhagen N DK-2200, Denmark; 5 Transgenic Mice Core Unit, Biotechnology Programme, CNIO, Madrid E-28029, Spain

## Abstract

Embryonic stem cells (ESCs) represent a transient biological state, where pluripotency is coupled with fast proliferation. ESCs display a constitutively active DNA damage response (DDR), but its molecular determinants have remained elusive. Here we show in cultured ESCs and mouse embryos that H2AX phosphorylation is dependent on Ataxia telangiectasia and Rad3 related (ATR) and is associated with chromatin loading of the ssDNA-binding proteins RPA and RAD51. Single-molecule analysis of replication intermediates reveals massive ssDNA gap accumulation, reduced fork speed and frequent fork reversal. All these marks of replication stress do not impair the mitotic process and are rapidly lost at differentiation onset. Delaying the G1/S transition in ESCs allows formation of 53BP1 nuclear bodies and suppresses ssDNA accumulation, fork slowing and reversal in the following S-phase. Genetic inactivation of fork slowing and reversal leads to chromosomal breakage in unperturbed ESCs. We propose that rapid cell cycle progression makes ESCs dependent on effective replication-coupled mechanisms to protect genome integrity.

## Abstract (web summary)

In fast proliferating embryonic stem cells (ESC) the DNA damage response is activated by mechanisms that are as yet elusive. Here, Ahuja *et al.* link the DNA damage response to replication stress in mouse ESCs, caused by a short G1 phase, and propose fork remodelling as maintaining genome stability in embryos.

## Graphical abstract

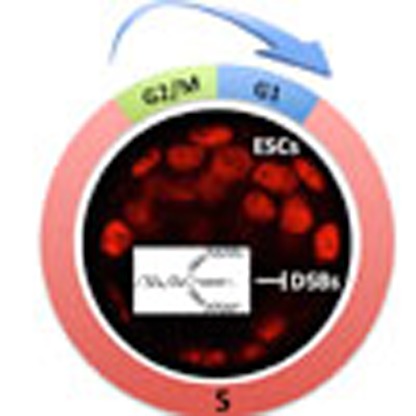

The earliest stages of mammalian embryogenesis are dedicated to the rapid division of totipotent cells in the morulas, later organized in the inner cell mass (ICM) of the blastocysts, from which embryonic stem cells (ESCs) are derived. Although ESCs can be maintained for long periods in cell culture, the equivalent cells in the ICM exist only transiently (5–6 cell divisions), in a period preceding the onset of differentiation^1^. Several reports have compared the DNA damage response (DDR) in ESCs with that in differentiated cells upon exogenous genotoxic insults^2^,^3^,^4^,^5^. However, little is known about how ESCs cope with endogenous stress that may arise during early embryogenesis. In ESCs, active proliferation needs to be compatible with accurate and complete DNA replication, to execute the developmental programme in a timely manner, without compromising genome stability in the embryo. Unexpectedly, it was previously reported that this stage is associated with constitutive DDR activation (phosphorylation of the histone variant H2AX, or γH2AX)^6^,^7^,^8^,^9^. However, as γH2AX appearance is not dependent on the activity of the apical checkpoint kinase ATM, and is not associated with the double-strand break (DSB) marker 53BP1, γH2AX was attributed to undefined peculiarities of ESC chromatin structure. Hence, the possible presence of DNA damage in these cells has remained controversial and elusive.

An important feature that sets ESCs apart from mature cells is the different organization of the cell cycle^10^,^11^. Differentiated cells spend a relatively large proportion of their time in the gap phases (G1 and G2) and lesser time in the S-phase. Conversely, asynchronously growing ESCs have remarkably short gap phases and spend most of their time in the S-phase, although the time spent for genome duplication is not significantly different from that in somatic cells^12^. In line with their high proliferative capacity, most positive cell cycle regulators and DNA replication factors (for example, CDC25A, CDC6, cyclins and so on) are extremely abundant in ESCs compared with mouse embryonic fibroblasts (MEFs)^13^ and their levels drastically drop down on ESC differentiation^14^. This unusual cell cycle control is orchestrated by key stem cell factors^5^,^15^ and was shown to be essential to maintain pluripotency in ESCs^16^,^17^. Furthermore, ESCs are reported to have a compromised G1–S checkpoint^2^,^18^. The tumour suppressor protein retinoblastoma, which is required for prevention of aberrant G1–S progression, thereby preventing damaged DNA from being replicated, is active in MEFs, but inactive in ESCs^19^. In principle, this could enable ESCs to enter S-phase in the presence of unrepaired damage.

In this study, we have investigated the molecular determinants of the constitutive DDR activation observed in ESCs. We found several unexpected markers of genotoxic stress during ESC replication, which explain activation of the Ataxia telangiectasia and Rad3 related (ATR) pathway and result from cell cycle adaptations of ESCs, specifically from the fast transition through the G1 phase. We propose that fast proliferating ESCs lack effective mechanisms to delay G2/M and G1/S transitions on incomplete replication, but effectively protect genome integrity by replication-coupled mechanisms. As hyperproliferation and replication problems in adult stem cells have been recently linked to cancer onset and stem cell attrition^20^,^21^, molecular mechanisms related to those described here in early embryogenesis may underlie key causative events in human disease.

## Results

### RPA/RAD51 chromatin loading in early embryogenesis

**Figure 1:**
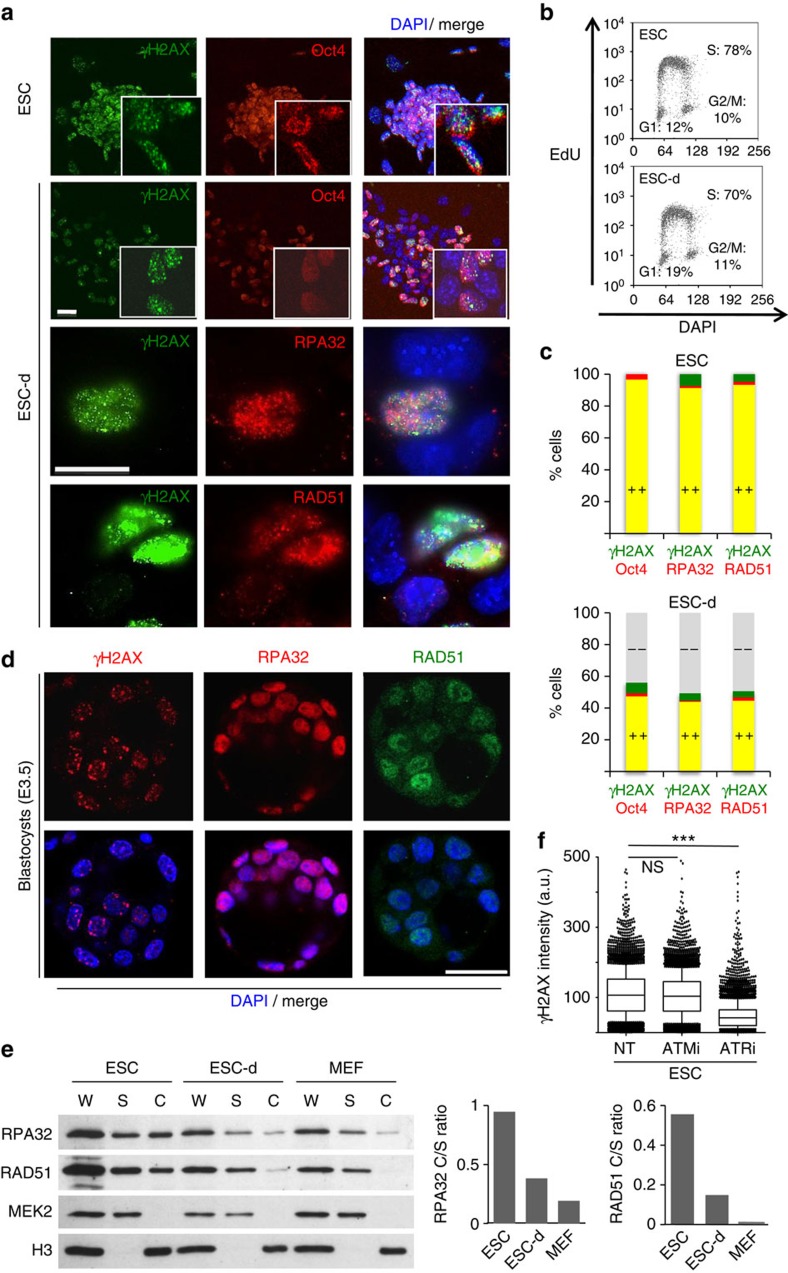
Replication stress markers are present *in vitro* in ESCs and *in vivo* in ICM cells. (**a**) Immunofluorescence (IF) staining of an embryonic stem cell (ESC) colony and partially differentiated ESCs (*ESC-d*; 3d from LIF removal) for the stem cell marker Oct4, the DNA damage marker γH2AX and chromatin-bound ssDNA-binding proteins RPA32 and RAD51. Scale bars, 25 μm. (**b**) FACS analysis of EdU incorporation and DNA content (DAPI) in ESC and ESC-d. The percentage of cells in the different cell cycle phases is indicated. (**c**) Quantification of double IF-stainings displayed in a) in ESC and ESC-d. A minimum of 150 cells were scored in each double staining. (**d**) IF staining for γH2AX, RPA32 and RAD51 of E3.5 blastocysts. Number of embryos analysed per staining was 12, 11 and 9, respectively. Representative images are shown (see also Supplementary Figs 2 and 3). Scale bar, 25 μm. (**e**) Immunoblot detection of the indicated proteins after biochemical fractionation performed on ESC, ESC-d and mouse embryonic fibroblasts (MEFs). Cytosolic kinase MEK2 and chromatin-bound H3 serve as fractionation controls. Original films were scanned at high resolution and the intensity of immunoblot signals was quantified with ImageJ. C, chromatin-enriched fraction; S, soluble fraction (includes cytosol and abundant nucleosoluble proteins); W, whole-cell extract. Histograms represent chromatin/soluble (C/S) ratios for RPA32 (left) and RAD51 (right). (**f**) FACS-based quantification of γH2AX staining in ESCs on mock, ATM inhibitor (ATMi) or ATR inhibitor (ATRi) treatment. All these experiments were performed in duplicate. a.u., arbitrary units.

To shed light on the molecular determinants of γH2AX formation in ESCs, we tested whether other markers of genotoxic stress are also detectable in these cells. We confirmed that, unless irradiated, ESCs are devoid of 53BP1 foci (Supplementary Fig. 1a)^9^, but also found that γH2AX in unperturbed ESCs is invariably associated with extensive chromatin loading of RPA32 and RAD51, two single-stranded DNA (ssDNA)-binding proteins involved in recombinational mechanisms at DSBs and stalled forks^22^. All three markers are lost simultaneously on induction of differentiation by removal of leukaemia inhibition factor (LIF)^23^, as soon as the stem cell marker Oct4 is lost from the differentiating cells (ESC-d) and while cells are still largely undergoing active proliferation (Fig. 1a–c). Similar observations were made in different ES cell lines, under different cell culture conditions (Supplementary Fig. 1b,c) and were confirmed *in vivo* by staining of pre-implantation mouse embryos at the morula and blastocyst stage (Fig. 1d; Supplementary Figs 2 and 3). Biochemical fractionation confirmed extensive chromatin loading of RPA and RAD51 in ESCs, compared with the levels observed in differentiating and differentiated cells (Fig. 1e). Furthermore, ATM inhibition by KU55933 reduced infrared-induced γH2AX in ESCs, but had no effect on endogenous γH2AX levels in these cells (Fig. 1f; Supplementary Fig. 1d,e), as previously reported^6^. Conversely, 8-h treatment with ATR inhibitors (ETP-46464 or VE-821) did not affect ESC proliferation, but markedly reduced endogenous γH2AX (Fig. 1f; Supplementary Fig. 1f–h). Altogether, these data strongly suggest that ESCs experience *bona fide* replication stress, which is rapidly suppressed on onset of differentiation.

### ssDNA and fork remodelling in ESCs until differentiation onset

**Figure 2:**
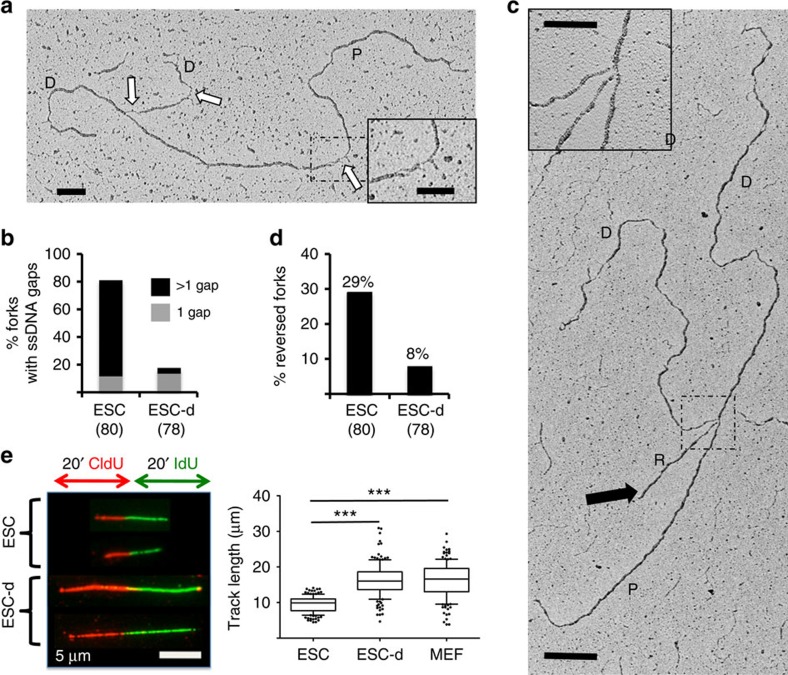
ESCs display massive accumulation of ssDNA gaps, reduced fork speed and frequent fork reversal. (**a**,**c**) Electron micrographs of representative replication forks from ESCs, with indicated parental (P) and daughter (D) duplexes. White arrows indicate ssDNA gaps; black arrow points to the regressed arm (R) of a reversed fork. Insets: (**a**), a magnified ssDNA gap; (**c**), the four-way junction at the reversed fork. Scale bar, 500 bp (=217 nm), 200 bp in inset. (**b**) Frequency of replication forks isolated from ESCs and differentiating ESCs (ESC-d) with the indicated number of ssDNA gaps. (**d**) Frequency of reversed replication forks isolated from ESC and ESC-d. The number of replication intermediates analysed is indicated in parentheses. Similar results were obtained in an independent experiment. (**e**) DNA fibre spreading of ESC, ESC-d and MEF. CldU/IdU-containing tracts were immunostained in red and green, respectively. Two representative fibres are shown for ESCs and ‘Differ. ESCs'. The IdU replicated track length was computed using Mann–Whitney test.

As RPA32/RAD51 staining are suggestive of ssDNA accumulation, we applied an established single-molecule approach to stabilize replication intermediates in cultured cells and visualize them by electron microscopy^24^. Replication intermediates in ESCs display a striking accumulation of ssDNA gaps, with multiple gaps present in over 60% of the forks (Fig. 2a,b), frequently also on parental DNA (Fig. 2a; Supplementary Fig. 4a). As for other experimental conditions associated with template discontinuities^25^,^26^, ssDNA gap accumulation is accompanied by frequent replication fork reversal (Fig. 2c,d; Supplementary Fig. 4b), a protective transaction previously described at replication forks challenged by cancer chemotherapeutic treatments^26^,^27^,^28^. Strikingly, both ssDNA accumulation and fork reversal are rapidly and markedly suppressed on induction of differentiation (Fig. 2b,d). Incomplete Oct4 loss at this early time point of differentiation is likely to account for the residual levels of ssDNA and reversed forks observed (Fig. 2b,d; Supplementary Fig. 4c). These marks of replication stress in ESCs are associated with a marked reduction in replication fork speed measured by DNA fibre spreading compared with differentiating and differentiated counterparts (Fig. 2e). Reduced fork speed in ESCs was also observed with shorter labelling times and on inhibition of origin firing (Supplementary Fig. 4d–f), excluding that these data reflect frequent fork termination due to reduced replicon size in these cells^29^.

### Replication stress in ESCs does not perturb mitosis

**Figure 3:**
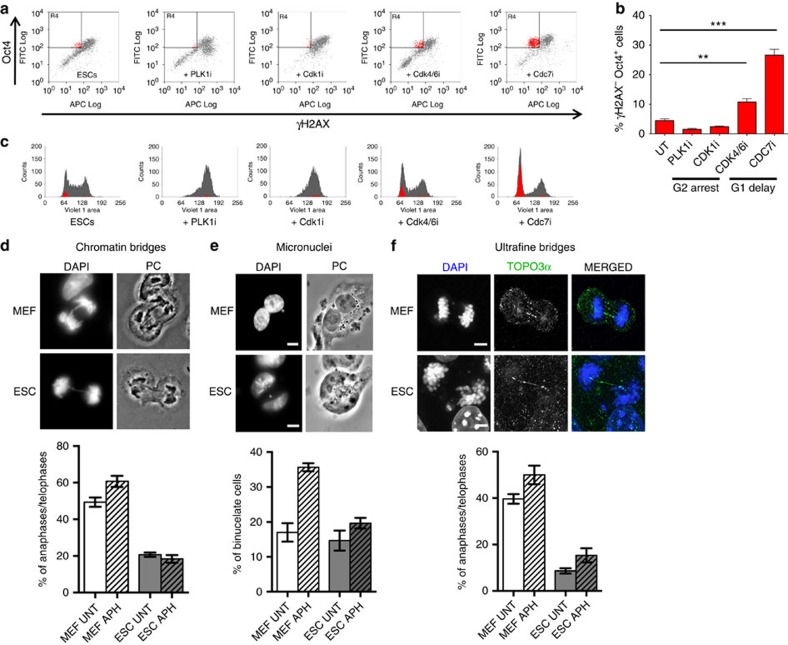
Delaying the G1/S and not the G2/M transition suppresses H2AX phosphorylation in ESCs. (**a**) FACS analysis of Oct4 and γH2AX in ESCs on the indicated treatments. PLK1i (BI-6727) and CDK1i (RO-3306) treatments delay progression through G2/M, while CDK4/6i (LY-2835219) and CDC7i (PHA-767491) treatments delay S-phase entry. The top-left quadrant identifies the subpopulation of stem cells (Oct4^+^) that display negative γH2AX staining. (**b**) Student's *t*-test of the subpopulation of Oct4^+^ γH2AX^−^ cells (red) identified in **a**, across three independent experiments. Error bars indicate s.d. **P<0.005, ***P<0.0005. (**c**) Cell cycle distribution, assessed by FACS-based DNA content (DAPI), of the samples in **a**. The total population is displayed in grey and the subpopulation of Oct4^+^ γH2AX^−^ in red. Where present, Oct4^+^ γH2AX^−^ cells are invariably detected in G1. (**d**–**f**) Representative images and graphical distribution of untreated (UNT) and aphidicolin (APH)-treated MEFs and ESCs displaying different mitotic defects (**d**, chromatin bridges; **e**, micronuclei; **f**, ultrafine bridges). Scale bars, 10 μm. The histograms indicate mean and standard deviations from three independent experiments.

While addressing the molecular determinants of ssDNA accumulation in ESCs, we tested several hypotheses, possibly associated with peculiarities of stem cell biology, using FACS/IF-based assessment of γH2AX on genetic or chemical perturbations (Supplementary Figs 5 and 6). In this way, we excluded that the DDR in ESCs specifically reflects oxidative stress or nucleotide shortage (Supplementary Fig. 5a–f), previously reported to mediate replication stress in other experimental systems^30^,^31^,^32^. Furthermore, two different transcription inhibitors failed to reduce γH2AX levels in ESCs (Supplementary Fig. 5g–j); thus, although replication/transcription interference may contribute to replication stress in ESCs, it does not seem the key underlying mechanism of constitutive DDR activation in early embryogenesis. We also excluded that ssDNA gap accumulation results from active demethylation—a base excision repair-associated process mediating the epigenetic reprogramming in ESCs^33^—as inactivation of key enzymatic activities in this process also had no detectable effect on the observed γH2AX levels (Supplementary Fig. 6a–e). Although partial and combined contributions of the above mechanisms cannot be formally ruled out, we next focused on other potential ESC-specific sources of genotoxic stress. As both ssDNA accumulation and fork reversal were recently reported on induction of multiple replication rounds within a given S-phase^25^, we hypothesized that replication stress in unperturbed ESCs may reflect their peculiar cell cycle progression^10^ with replication rounds in rapid succession—spaced by short gap phases^12^—and the absence of a functional G1/S checkpoint^2^,^18^. To test the hypothesis that specific cell cycle constraints may underlie the observed marks of replication stress, we delayed progression through G2/M- or G1/S-phase transitions and tested whether those cells experiencing a prolonged G1 or G2 phase would detectably suppress the DDR (γH2AX−), while retaining stem cell properties (Oct4+). G2/M arrest was achieved by a PLK1 (BI-6727) or a CDK1 inhibitor (RO-3306), while G1 delay was induced by a CDK4/6 (LY-2835219) or a CDC7 inhibitor (PHA-767491). Only a small fraction of cycling ESCs displayed low levels of γH2AX while quickly transitioning through G1 (Fig. 3a–c). The proportion of γH2AX− Oct4+ cells was not increased by extending the length of the G2 phase (Fig. 3a–c). In contrast, a delay in the G1/S transition did result in the accumulation of γH2AX− Oct4+ cells, particularly evident in the cells effectively arrested with unreplicated DNA on CDC7 inhibition (Fig. 3a–c). These data, combined with previous observations (Figs 1 and 2), suggest that accumulated replication stress in ESCs is unlikely to be addressed in mitosis, but can instead be dealt within the following G1, if cells are allowed to spend sufficient time in this phase. We thus tested directly whether cycling ESCs display aberrant mitotic structures that are typically associated with unfinished replication in somatic cells, such as chromatin bridges, micronuclei and ultrafine DNA bridges^34^. Surprisingly, despite markedly lower levels of replication stress marks than in ESCs (Figs 1 and 2), MEFs displayed higher endogenous levels of unresolved mitotic structures, which as expected were further induced by aphidicolin addition, an established treatment to challenge replication completion (Fig. 3d–f). Conversely, albeit displaying and signalling marked features of replication stress, unperturbed ESCs showed limited numbers of mitotic defects, which were only marginally increased on aphidicolin treatments (Fig. 3d–f). These surprising data strongly suggest that the mitotic process in ESCs is tuned to tolerate elevated levels of replication stress, as those reported above for cultured ESCs and mouse blastocysts.

### A short G1 causes replication stress in stem and mature cells

**Figure 4:**
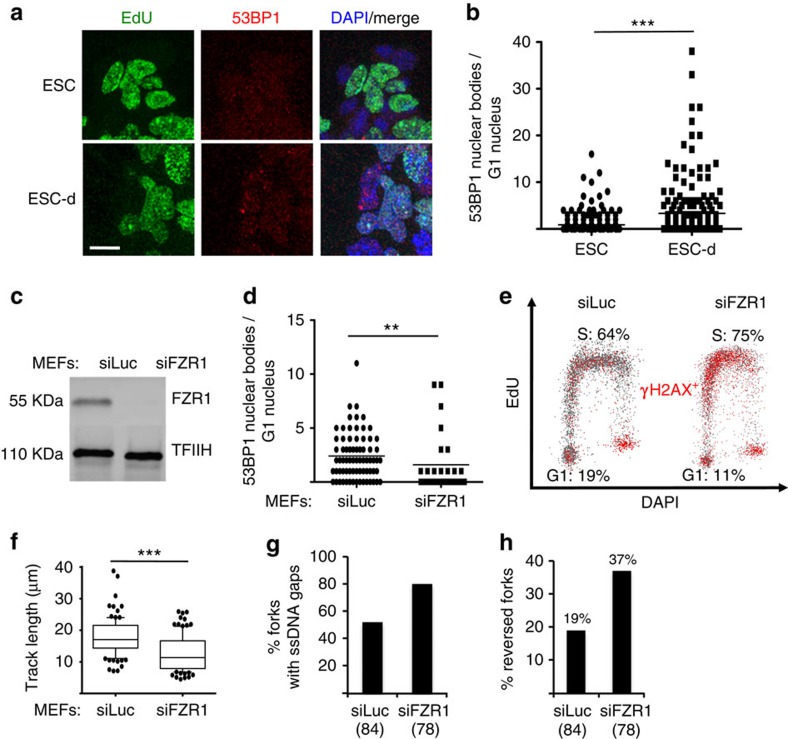
A shortened G1 phase impairs 53BP1 nuclear body formation and causes replication stress in the following S-phase. (**a**) Representative IF images of ESC and ESC-d stained with EdU and 53BP1. ESC differentiation detectably increase 53BP1 nuclear signals in EdU-negative cells. Scale bar, 25 μm. (**b**) Microscopically discernible 53BP1 nuclear bodies (NBs) in G1 nuclei (as identified by low DAPI and low EdU signals) were quantified in asynchronous populations of ESC and ESC-d by software-assisted segmentation and feature extraction (Supplementary Fig. 6f). Comparable results were observed in two additional, independent experiments. (**c**) Western blot analysis of FZR1 levels in mock-depleted (siLuc) and FZR1-depleted (siFZR1) MEFs, 96 h after siRNA transfection. TFIIH is used as loading control. (**d**) Microscopically discernible 53BP1 NBs specifically in G1 nuclei of asynchronous populations of mock- and FZR1-depleted MEFs were quantified as in **b**. (**b**,**d**) **P<0.005, ***P<0.0005 (Mann–Whitney test) (**e**) FACS analysis for DNA synthesis (EdU incorporation), DNA content (DAPI) and DDR activation (γH2AX) in mock-depleted and FZR1-depleted MEFs. Plots depict EdU incorporation versus DAPI in both populations. γH2AX positive cells are depicted in red. Cell populations in G1 and S-phase are also depicted. (**f**) DNA fibre analysis to assess fork progression in mock-depleted and FZR1-depleted MEFs. The IdU replicated track length was computed using Mann–Whitney test. (**g**,**h**) EM-based assessment of the percentage of replication intermediates containing ssDNA gaps (**g**) and the percentage of reversed forks (**h**) in genomic DNA extracted from mock-depleted and FZR1-depleted MEFs.

Differently from the G2/M-phase, prolongation of the G1 phase by two different inhibitors significantly reduced γH2AX signalling in ESCs (Fig. 3a–c). Intriguingly, the G1 phase was recently reported to allow the assembly of large 53BP1 foci (nuclear bodies, NBs) in somatic cells^35^. These specialized chromatin machineries in the G1 phase are clearly distinct from small DNA damage-induced 53BP1 foci and were suggested to shield residual DNA damage or unreplicated regions from the previous S-phase, possibly assisting their repair before or during a new replication round^35^. We thus tested whether a prolonged G1 phase on ESC differentiation could be linked to the presence of 53BP1 NBs in this cell cycle phase. To achieve this, we combined quantitative image-based cytometry^36^—which identifies G1 cells in asynchronous cell populations by low DNA content (DAPI) and lack of EdU incorporation (Supplementary Fig. 6f)—with quantitative measurements of focal protein accumulation^37^,^38^. This approach indeed revealed that, in unperturbed conditions, ESCs in G1 are practically devoid of 53BP1 NBs. However, shortly after induction of differentiation (ESC-d), along with prolongation of this cell cycle stage, G1 cells accumulated 53BP1 NBs (Fig. 4a,b; Supplementary Fig. 6f). To further explore the hypothesis that a short G1 phase underlies constitutive DDR activation in ESCs, we performed the reciprocal experiment, shortening the G1 phase in differentiated cells (MEFs) and assessing markers of replication stress and DDR activation. Shortening of the G1 phase in differentiated mouse cells can be achieved by inactivation of the APC/C activator FZR1 (ref. 39). Indeed, small interfering RNA (siRNA)-mediated FZR1 depletion in MEFs reduced the fraction of G1 cells and concomitantly decreased the number of 53BP1 NBs per G1 nucleus (Fig. 4c–e), thus phenocopying what we found in unperturbed ESCs with an intrinsically short G1 phase. Importantly, in these experimental conditions, S-phase cells displayed markedly increased γH2AX signalling, reduced replication fork progression by DNA fibre assays and higher levels of ssDNA gaps and reversed forks detected by our electron microscopy approach (Fig. 4e–h). Altogether, these results strongly suggest that a prolonged G1 phase and/or the associated assembly of 53BP1 NBs are required to avoid replication stress and to prevent the consequent activation of the ATR-checkpoint in the following S-phase.

### A prolonged G1 phase suppresses replication stress in ESCs

**Figure 5:**
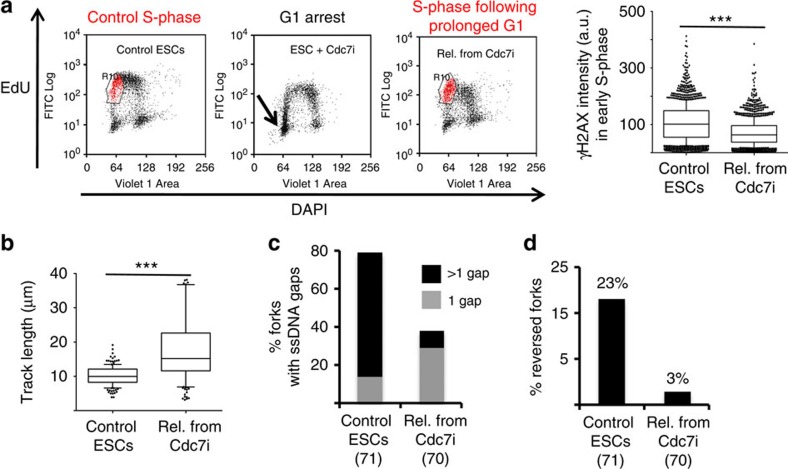
A prolonged G1 phase in ESCs suppresses replication stress in the following S-phase. (**a**) FACS analysis of EdU incorporation and DNA content (DAPI) in control ESCs, ESCs treated with CDC7i, and ESCs on release from a transient CDC7i arrest (PHA-767491, 10 μM, 8 h). The gated (red) subpopulation of cells in early S-phase was analysed quantitatively for γH2AX staining (a.u., arbitrary units). (**b**) IdU replicated track length, computed using Mann–Whitney test, in control ESCs and cells released from a transient CDC7i arrest. (**c**) Frequency of replication forks isolated from control ESCs and on release from a transient CDC7i arrest, displaying the indicated number of ssDNA gaps. (**d**) Frequency of reversed replication forks isolated from control ESCs and on release from a transient CDC7i arrest.

Prompted by the above evidence, we tested directly whether a transient G1 arrest in ESCs—by CDC7 inhibition (Fig. 3a)—would be sufficient to suppress the marks of replication stress in the following S-phase. On release from this transient G1 arrest, ESCs indeed displayed reduced γH2AX staining and a faster rate of replication fork progression (Fig. 5a,b). Strikingly, this transient delay in the G1/S transition was also sufficient to effectively suppress ssDNA accumulation and fork reversal on ESC replication intermediates *in vivo* analysed by electron microscopy (Fig. 5c,d). Taken together, these data strongly suggest that all unusual molecular hallmarks of ESC replication reflect the rapid succession of multiple replication rounds.

### Fork slowing and reversal protect ESCs from chromosome breaks

**Figure 6:**
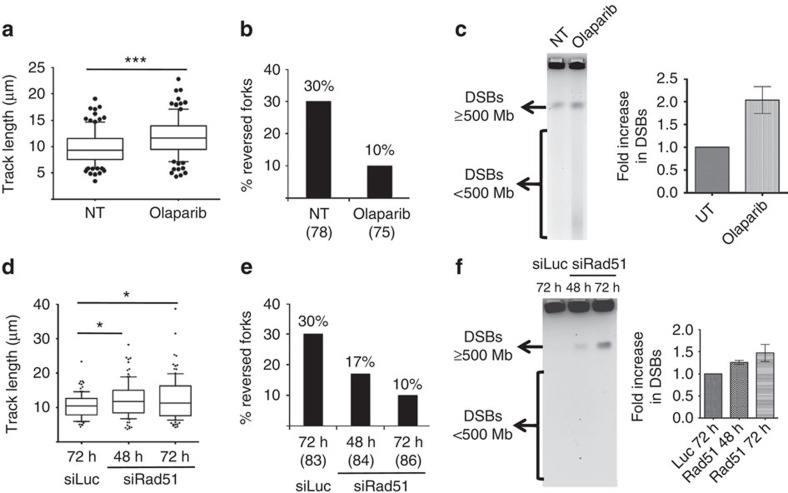
PARP and Rad51 are essential for replication fork protection in ESCs. (**a**,**d**) DNA fibre analysis of mock-treated ESCs, ESCs treated with Olaparib (**a**), mock-transfected ESCs and ESCs transfected with siRad51 for 48 and 72 h (**d**). The graphs show the statistical analysis of IdU replicated track length. (**b**,**e**) Frequency of reversed replication forks isolated from mock-treated ESCs and ESCs treated with Olaparib (**b**), mock-transfected ESCs and ESCs transfected with siRad51 for 48 and 72 h (**e**). (**c**,**f**) Pulse field gel electrophoresis (PFGE) to assess accumulation of DNA double-strand breaks (DSBs) in mock-treated ESCs, ESCs treated with Olaparib (**c**), mock-transfected ESCs and ESCs transfected with siRad51 for 48 and 72 h (**f**). DSBs equal to and above 500 Mb compact into a single band and DSBs smaller than 500 Mb can be detected as a smear as indicated alongside the gel. DSB (band plus smear) intensity from three independent experiments was quantified and plotted.

We and others have recently reported some genetic dependencies for replication fork slowing and reversal on genotoxic treatments of somatic cells, that is, Poly (ADP-ribose) polymerase (PARP) and the central recombinase RAD51 (refs 26, 28, 40). Therefore, we set out to test the physiological relevance of replication fork remodelling in the face of endogenous replication stress in otherwise unperturbed ESCs. Indeed, PARP inhibition by Olaparib in ESCs led to unrestrained fork progression (Fig. 6a) and to a marked (threefold) reduction in the frequency of reversed forks (Fig. 6b). Importantly, these defects were associated with detectable accumulation of small chromosomal fragments—likely resulting from frequent replication fork breakage (Fig. 6c)—and with defective completion of genome duplication (Supplementary Fig. 7a). Due to the abundance and the essential role of RAD51 in ESCs, its complete depletion by siRNA was difficult to achieve (Supplementary Fig. 7b). However, the observed reduction in RAD51 levels was sufficient to significantly increase fork speed and progressively impair efficient fork reversal (Fig. 6d,e), in keeping with recent evidence in somatic cells on genotoxic treatments^28^. Also in this independent condition of defective fork slowing and reversal, we observed significant and progressive accumulation of chromosomal breakage. The breaks occur while most cells are still actively proliferating (Supplementary Fig. 7c) and thus precede cell cycle arrest, which is expected on prolonged RAD51 depletion^41^. Taken together, these data suggest that replication fork slowing and reversal are essential in unperturbed ESCs to prevent the breakage of replicating chromosomes, which are challenged by endogenous replication stress.

## Discussion

**Figure 7:**
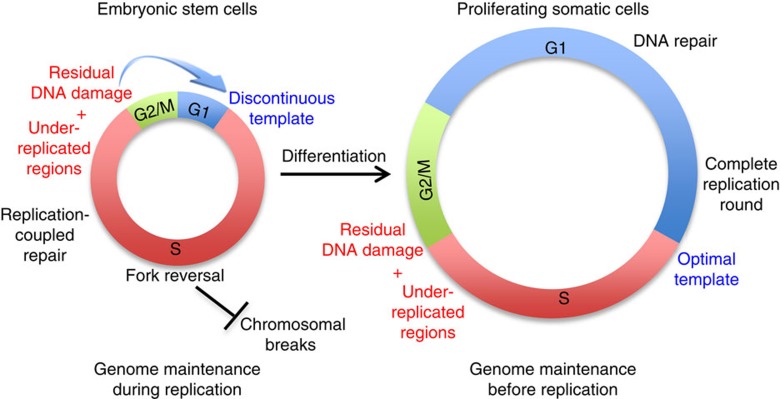
Model depicting differential control of genome stability in ESCs and proliferating somatic cells. Under-replicated regions and residual DNA damage are unavoidably present at the end of each S-phase in both ESCs and somatic cells. However, owing to the brief gap phases, ESCs channel a high number of these lesions in the following S-phase and protect genome integrity by extensive fork reversal and replication-coupled repair. Conversely, differentiated cells have prolonged gap phases, assemble 53BP1 NBs and repair most of these lesions before S-phase entry (see also Supplementary Fig. 7d).

The process of genomic duplication is usually challenged by under-replicated regions, which are frequently associated with late-replicating and difficult-to-replicate loci^34^,^42^. Thus, full genome replication often requires the delay of mitotic onset; furthermore, cells may eventually attempt segregation of partially replicated chromosomes, completing DNA synthesis in the following cell cycle^34^. Proliferating somatic cells take advantage of a prolonged G1 phase to complete genome duplication, using specialized mechanisms—such as the assembly of 53BP1 NBs^35^—that are proposed to mediate template quality control before the onset of a new replication round (Fig. 7). In line with this notion, we now report that shortening the G1 phase in differentiated cells is sufficient to impair the assembly of 53BP1 NBs and to induce the full set of unusual replication features detected in unperturbed ESCs. Conversely, prolongation of the G1 phase is sufficient to restore 53BP1 NBs assembly and to largely suppress replication stress marks in ESCs (Supplementary Fig. 7d). Thus, our data bring further support to the notion that 53BP1 NBs do represent specialized chromatin factories promoting DNA repair before or during a new replication round^35^, in response to both endogenous and exogenous sources of genotoxic stress (Supplementary Fig. 7d). The detailed molecular mechanisms underlying these DNA repair events will merit further investigation.

In contrary to differentiated cells, ESCs appear highly tolerant of endogenous and exogenous replication stress while undergoing mitosis. We found reduced levels of unresolved mitotic structures, despite obvious markers of endogenous replication stress being detected in these cells, even when exogenous stress was induced by aphidicolin treatment. It is likely that the type of DNA damage accumulated in these cells at the end of replication—mostly ssDNA gaps on replicated duplexes—may not interfere with spindle elongation and chromosome segregation and may thus be ineffective in activating cell cycle checkpoints. Whether specific molecular features of the mitotic apparatus actively promote cell division in ESCs even in the presence of residual stress will need further investigation. Besides this unrestrained transition through mitosis, a particularly short G1 phase is also somehow instrumental in ESCs maintaining self-renewal^16^,^17^. Furthermore, these cells lack the molecular mechanisms that delay S-phase entry in the presence of DNA damage^18^. In light of these combined observations, we propose that ESCs do not finalize a replication round before the onset of the following S-phase (Fig. 7). This leads to a discontinuous replication template and forces these cells to undergo simultaneous replication and repair synthesis. The abundance of simultaneous DNA synthesis events in these cells, exacerbated by the increased usage of replication origins^43^, may exhaust limiting factors for DNA synthesis and further affect the replication process^44^, contributing to the observed accumulation of ssDNA. In this respect, prolonging the G1 phase may assist the following replication round not only by providing more time for full genome duplication, but also by modulating the level of limiting replication factors and/or the number and distribution of licensed replication origins, thereby preventing accumulation of replication stress. In keeping with this, treating ESCs with Roscovitine—a CDK inhibitor known to limit origin activity—leads to a fivefold increase in the number of stem cells losing γH2AX, mostly residing within the G1 phase (Supplementary Fig. 7e,f).

Despite the short residence time in G1 phase and the associated consequences in terms of replication stress, we propose that ESCs can effectively tolerate this stress for those few cell division cycles that they undergo in nature, by exploiting replication-coupled control of genome stability. Unperturbed ESCs seem extremely efficient in promoting replication fork reversal, even in the absence of exogenous damage. In line with recent data obtained in somatic cells on genotoxic treatments^26^,^28^, we report here that the accumulation of reversed forks in ESCs depends on the key recombinase RAD51 and on PARP activity. Genetic inactivation of either factor in unperturbed ESCs impairs effective fork slowing and reversal, which rapidly results in chromosomal breakage. These results strongly suggest that controlled fork progression and remodelling play a pivotal role in protecting the integrity of replicating chromosomes in ESCs, which have extremely short gap phases and thus rely entirely on genome maintenance during replication (Fig. 7). Considering the abundance of RAD51 and its essential function in early embryogenesis^41^,^45^, it is tempting to speculate that the essential developmental role of RAD51 and possibly other homologous recombination factors reflects their extensive involvement in replication fork remodelling, to prevent chromosomal breakage during active proliferation in early embryogenesis. Conversely, the well-established role of HR factors in DSB repair may become more relevant in germ and somatic cells after programmed DSB formation and/or exposure to environmental clastogens. In this respect, as new molecular mechanisms modulating *in vivo* replication fork remodelling and restart are being uncovered^27^, it will be important to test their specific contribution to genome integrity in early embryogenesis. Interestingly, besides the G1 shortening induced in MEFs by FZR1 depletion, also the activation of oncogenes that accelerate the G1–S transition—that is, CyclinE^46^—induces in somatic cells molecular hallmarks of replication stress that are almost identical to those described here in ESCs, including frequent fork reversal. Somatic cells can tolerate CyclinE-induced replication stress for several cell cycles before inducing chromosomal breakage^47^. In this respect, our data in ESCs consolidate the emerging notion that fork reversal represents a widely used strategy for genome maintenance, in the face of different types of replication stress^27^. It is important to note that—besides the DNA damage tolerance mechanisms described here—genome maintenance in early embryogenesis is further supported by elimination of damaged cells by apoptosis, reportedly a very active process in this biological context^18^.

It will be crucial, albeit challenging, to extend these studies to adult stem cells (for example, hematopoietic stem cells), particularly in experimental conditions where they are actively induced to proliferate by specific stimuli^48^. This is particularly important in light of recent reports suggesting that replication stress contributes to aging and attrition of hematopoietic stem cells, upon physiological stimuli to proliferate^21^,^49^. Remarkably, the proliferative properties of different populations of adult stem cells have also been recently linked to the propensity of different tissues to undergo tumorigenesis, suggesting that replication problems in adult stem cells can be initiating events in cancer^20^. In light of these important findings, the working model described here for replication stress in early embryogenesis may prime new mechanistic studies on different populations of adult stem cells, especially under cancer-relevant experimental conditions. Similar mechanisms may also be relevant in the derivation of induced pluripotent stem cells, and may contribute to explaining the reported signs of replication stress and genome damage, which limit induced pluripotent stem cell derivation and raise concerns for their therapeutic applications^50^,^51^,^52^,^53^.

## Methods

### Cells and cell culture

E14, Stat3 and JM8 mouse ES cell lines, derived from the ICM of mouse blastocysts, were provided by Paolo Cinelli (Clinic for Trauma Surgery, University of Zurich), while immortalized MEFs were provided by F. Althaus (Institute of Pharmacology and Toxicology, University of Zurich). E14 were cultivated in ‘ESC medium'+LIF and differentiation was started by LIF removal. Stat3 and JM8 were cultivated in ‘N2B27 medium' (see composition below) +2i and differentiation was started by 2i removal. ESC medium: Dulbecco's Modified Eagle Medium (Sigma), 15% FBS (Gibco), 1 mM Sodium Pyruvate (Sigma), 1 × Non-Essential Amino Acids (Sigma), 1 × Penicillin-Streptomycin-L-Glutamine (Life Technologies), 0.1 mM β-Mercaptoethanol (Sigma). N2B27 medium: 1:1 Neurobasal Medium:Dulbecco's Modified Eagle Medium/F-12 (Life Technologies), 1 × Penicillin-Streptomycin-L-Glutamine (Life Technologies), 1 × N2-Supplement (Life Technologies), 1 × B27 Supplement (Life Technologies), 0.05 mM 2-Mercaptoethanol (Life Technologies). LIF: 1,000 U ml^−1^ (Millipore). 2i: 1 μM PD0325901 (Stemgent), 3 μM CHIR99021 (Stemgent). ESCs were cultivated on feeder cells (MEFs inactivated with 10 mg ml^−1^ mitomycin C) for at least two passages before performing experiments. ESCs were then separated from feeder cells by trypsinization and centrifugation, and grown on gelatinized tissue culture dishes (0.1% Gelatin from porcine skin, Sigma).

### Mouse morula and blastocyst isolation and staining

Mice used in these experiments were hosted at the CNIO animal facility and maintained in SPF conditions. All procedures were approved by the Ethics Committee of the Instituto de Salud Carlos III and the competent organism of the Comunidad Autonoma de Madrid. 6- to 8-week-old B6/CBA or CD1 females were induced to superovulate by intraperitoneal injection of 5 IU pregnant mare serum gonadotropin (PMSG) followed 48 h later by 5 IU of human chorionic gonadotropin (HCG). Matings with fertile males of the same genetic background were set up from the day of human chorionic gonadotropin administration. Morulas were collected at day E2.5 and blastocysts at day E3.5 of embryonic development. In all cases, embryos were collected in M2 medium (Sigma). After removal of the zona pellucida with acid Tyrode's solution (Sigma), embryos were fixed with 4% paraformaldehyde, permabilized with 0.25% Triton X-100 in PBS, blocked and incubated with primary antibodies diluted in blocking solution (0.1% BSA, 0.01% Tween 20, 2% normal donkey serum) overnight at 4 °C, washed and incubated in secondary antibodies for 1 h at room temperature. Stained embryos were placed in a drop of blocking solution containing 4 μg ml^−1^ DAPI on a glass-bottom dish, covered with mineral oil and kept at 4 °C until confocal microscopy analysis in a Leica TCS SP5 microscope equipped with objective HCX PL APO CS 63 × Water (NA 1.2). Z stacks were taken with a 1–2 μm step size. Images were processed using ImageJ.

The following primary antibodies were used: γH2AX (Millipore, 05–636) 1:500; RPA2, Cell Signalling, 2208S) 1:100; RAD51 (Santa Cruz Biotechnology, sc-8349) 1:250. Secondary antibodies: Alexa Fluor 555 Donkey anti-Mouse (Life Technologies, A31570); Alexa Fluor 594 Donkey anti-Rat (Life Technologies, A21209); Alexa Fluor 488 Donkey anti-Rabbit (Life Technologies, A21206) 1:500.

### Epifluorescence and confocal microscopy

For immunofluorescence, cultured ES cells were fixed with 4% formaldehyde/PBS, permeabilized with 0.25% Triton X-100 and stained for γ-H2AX (1:500), Oct4 (1:500), RPA (1:100), Rad51 (1:250), 53BP1 (1:500) and EdU as indicated, detected by appropriate secondary antibodies (Alexa Fluor, 1:500) or Click-IT reaction (Life Technologies), and mounted with Vectashield (Vector Laboratories). 0.1% PBST (0.1% Tween in 1 × PBS) was used for washes after primary and secondary antibody incubations. Images were acquired at × 63 on a Leica DMRB microscope or on a Leica TCS SP5 confocal microscope,

For quantitative image analyses (Fig. 4b,d and Supplementary Fig. 6f), automated wide-field microscopy was performed on a Leica DMI 6000 inverted microscope equipped with a motorized stage, a Tri-band bandpass filter (DAPI/FITC/TX; BP387/11/BP 494/20/BP 575/20) and a 12-bit monochrome EMCCD camera (Leica DFC 350 FX, 1,392 × 1,040 pixels, 6.4 μm pixel size). All images were acquired under non-saturating conditions with a HCX PLAN APO × 40 (NA 1.25–0.75) oil objective. Unbiased, automated image acquisition was performed with the Leica Matrix Screening Software. All images were imported to the Olympus ScanR Image Analysis Software Version 2.5.1, a dynamic background correction was applied, and nuclei segmentation was performed using the integrated EdgeDetection module. Focal accumulations of 53BP1 and γH2AX were quantified by using the integrated SpotDetector module. Colour-coded scatter plots of asynchronous cell populations were generated with Spotfire data visualization software (TIBCO). *P* values (Fig. 4b,d) were calculated by Mann–Whitney test.

### Transfections and treatments

For knockdown experiments, cells were transfected 24–96 h before sample collection with the indicated siRNA using RNAiMax transfection reagent (Life Technologies) according to the manufacturer's instructions: siLUC (100–200 nM; 5′-GGUACGCGGAAUACUUCGAdTdT-3′), siFZR1, siRad51, siAPE1, siTDG (100–200 nM siGENOME SMARTpool Dharmacon). The following reagents were used to treat ES cells for the indicated time at the indicated final concentrations before collection: ATM inhibitor (KU55933, Kudos; 8 h, 10 μM); ATR inhibitor (ETP-46464, provided by O. Fernandez-Capetillo, CNIO, Madrid; 8 h, 5 μM; VE-821, Selleckchem; 8 h, 10 μM); PARP inhibitor (Olaparib, Selleckchem; 24 h, 10 μM); Reducing/scavenging agent: (N-acetylcysteine, Sigma; 10 h, 10 mM); Transcription inhibitors (Cordycepin, Sigma; 100 min, 50 μM; Alpha-amanitin, kindly provided by P. Janscak, 3–6 h, 20 μM); Ape1 inhibitor (Methoxyamine hydrochloride, Sigma; 10 h, 1 μM); CDK4/6 inhibitor (LY2835219, Selleckchem; 4 h, 1 μM); Cdc7 inhibitors (PHA-767491, Sigma; 8 h, 10 μM; XL-413, kindly provided by C. Santocanale, 4 h, 10 μM); CDK1 inhibitor (RO-3306, Sigma; 10 h, 10 μM); PLK inhibitor (BI-6727, Selleckchem; 4 h, 500 nM); Nucleosides (EmbryoMax, Millipore; 24 h, 5 ×). Roscovitine (Seliciclib, Selleckchem; 8 h, 20 μM).

### Flow cytometry

For flow cytometric analysis for γ-H2AX/EdU/DAPI, cells were labelled for 30 min with 10 μM EdU, harvested and fixed for 15 min with 4% formaldehyde/PBS. Cells were washed with 1% BSA/PBS, pH 7.4, permeabilized with 0.5% saponin/1% BSA/PBS and stained with anti-γ-H2AX antibody (#05–636; EMD Millipore) for 2 h, followed by incubation with a suitable secondary antibody for 30 min. Incorporated EdU was labelled according to the manufacturer's instructions (#C-10425; Life Technologies). For flow cytometric analysis for γ-H2AX/Oct4/DAPI, cells were fixed and permeabilized as described above, followed by incubation with antibodies against γ-H2AX (#9718; Cell Signalling Technology) and Oct4 (#611203, BD Transduction Laboratories) and suitable secondary antibodies. In both assays, DNA was stained with 1 μg ml^−1^ DAPI. Samples were measured on a Cyan ADP flow cytometer (Beckman Coulter) and analysed with Summit software v4.3 (Beckman Coulter). For statistical analyses, Mann–Whitney test was applied to compute if differences in signal intensities were significant using Prism (GraphPad Software).

### Western blotting

Cells were collected and either snap frozen in liquid nitrogen or immediately lysed using 2 × Laemmli buffer. Protein amounts were normalized using known concentrations of BSA and protein absorbance was measured using Cary 60 Spectrophotometer technology. SDS-gels were run at 15–18 mA and proteins were either wet-blotted overnight (30 V, 4 °C) or for 2 h (100 V, 4 °C) on Hybond ECL transfer membrane (GE Healthcare). Membranes were blocked in 2% ECL Advance Blocking Reagent (GE Healthcare) in 0.1% TBST (1 × TBS supplemented with 0.1% Tween 20) for at least 30 min and incubated with primary antibodies overnight at 4 °C or at room temperature for 4 h in blocking solution and secondary antibodies were added for 1 h at room temperature (in blocking solution). Membranes were washed three times with 0.1% TBST, 10' each, after primary and secondary antibody incubations and detected with ECL detection reagent (GE healthcare). Differences in protein levels were normalized against the loading control and the signal intensity was quantified using ImageJ. For complete, uncropped blots with size markers, see Supplementary Fig. 8.

### Pulse field gel electrophoresis

ES cells were transfected or treated as indicated and cells were harvested by trypsinization. Agarose plugs of 2.5 × 10^5^ cells were prepared in a disposable plug mold (Bio-Rad Laboratories). Plugs were then incubated in lysis buffer (100 mM EDTA, 1% (wt/vol) sodium lauroyl sarcosinate, 0.2% (wt/vol) sodium deoxycholate, and 1 mg ml^−1^ proteinase K) at 37 °C for 72 h. Plugs were then washed four times in 20 mM Tris-HCl, pH 8.0 and 50 mM EDTA before loading onto an agarose gel. Electrophoresis was performed for 21 h at 14 °C in 0.9% (wt/vol) Pulse Field Certified Agarose (Bio-Rad Laboratories) containing Tris-borate/EDTA buffer in a pulse field gel electrophoresis (PFGE) apparatus (CHEF DR III; Bio-Rad Laboratories), according to the following protocol (block I: 9 h, 120° included angle, 5.5 V cm^−1^, 30–18 s switch; block II: 6 h, 117° included angle, 4.5 V cm^−1^, 18–9-s switch; block III: 6 h, 112° included angle, 4.0 V cm^−1^, 9–5 s switch). The gel was then stained with ethidium bromide and analysed by the AlphaImager system (ProteinSimple). Relative DNA DSB) levels were assessed by comparing DSB signals for each treatment to the background levels observed in untreated conditions using ImageJ.

### Antibodies

The following primary antibodies were used: γ-H2AX (#05–636; EMD Millipore), 53BP1 (ab21083; Abcam), Oct3/4 ((#611203; BD Transduction Laboratories), RPA (NA19L; Calbiochem), Rad51 (sc-8349; Santa Cruz Biotechnology, Inc.), GAPDH (#AB2302; Millipore), Fzr1 (ab3242; Abcam), RNA pol II (provided by Pavel Janscak), Ape1 (NB100–116; Novus), Tdg (provided by P. Schär), hTOPO3α (IE3; a kind gift from Aventis Pharma). For chromatin fractionation, Rad51(sc-8349; Santa Cruz Biotechnology, Inc.), RPA32 (2208S; Cell Signaling), MEK2 (610235; BD Transduction Laboratories), Histone-H3 (ab1791; Abcam). The secondary antibodies used were Alexa Fluor conjugates (Alexa Fluor 488, 594 and 647; Life Technologies) for flow cytometry and immunofluorescence and anti-rabbit and anti-mouse ECL (GE Healthcare) for western blotting.

### DNA fibre analysis

Cells were sequentially pulse-labelled with 30 μM CldU and 250 μM IdU for 20 min (15 min where indicated) each and harvested. Cells were then lysed and DNA fibres stretched onto glass slides by tilting them. The fibres were then denatured with 2.5 M HCl for 1 h, washed with PBS and blocked with 0.2% Tween 20 in 1% BSA/PBS. CldU and IdU tracks were detected with anti-BrdU antibodies recognizing CldU (ab6326; Abcam) and IdU (347580; BD), respectively, and appropriate secondary antibodies. Images were acquired with a microscope (model DMRB; Leica) equipped with a camera (model DFC360 FX; Leica). Images were taken at × 63 using Leica Application Suite 3.3.0. CldU and IdU tract lengths were measured using ImageJ. For statistical analyses, Mann–Whitney test was applied to compute if differences in tract lengths were significant using Prism (GraphPad Software).

### Electron microscopy analysis of DNA replication intermediates in mouse cells

*In vivo* psoralen cross-linking, isolation of total genomic DNA and enrichment of the replication intermediates and their electron microscopy visualization were performed as described^22^. In brief, cells were harvested, and genomic DNA was cross-linked by two rounds of incubation in 10 μM 4,5′,8-trimethylpsoralen, followed by 3 min of irradiation with 366 nm ultraviolet light. Cells were lysed, and genomic DNA was isolated from the nuclei by proteinase K digestion and phenol–chloroform extraction. Purified DNA was digested QJ;with PvuII-HF and replication intermediates were enriched on a BND cellulose column. Electron microscopy samples were prepared by spreading the DNA on carbon-coated grids and visualized by platinum rotary shadowing. Images were acquired on a transmission electron microscope (G2 Spirit; FEI Tecnai) and analysed with ImageJ (National Institutes of Health).

### Chromatin fractionation

ESCs, differentiating ESCs and MEFs were subjected to biochemical fractionation according to a standard protocol^54^. The distribution of proteins between equivalent amounts of the soluble and chromatin-enriched fractions was analysed by immunoblot.

### Analysis of mitotic defects

Cells were inoculated on poly-L-Lysine coated glass slides (SIGMA-Aldrich) and grown for 16 h in the absence or in the presence of 0.3 μM Aphidicolin. For ‘micronuclei' cells were additionally treated for 6 h with 5 μg ml^−1^ Cytochalasin-B before fixation. For ‘chromatin bridges' and ‘micronuclei', cells were fixed with 4% paraformaldehyde for 20 min and permeabilized with 0.5% Triton X-100 in PBS for 1 h. For ‘ultrafine bridges', cells were fixed using 4% paraformaldehyde, 0.5% Triton X-100 in PBS for 20 min. Slides were saturated using BSA 3%, Triton X-100 0.5% in PBS for a minimum of 2 h. Slides were then mounted in Vectashield mounting medium containing DAPI. The number of chromatin bridge-containing Anaphase/Telophase cells, micronuclei-containing bi-nucleated cells or UFB-containing Anaphase/Telophase cells was scored manually.

## Additional information

**How to cite this article:** Ahuja, A. K. *et al.* A short G1 phase imposes constitutive replication stress and fork remodelling in mouse embryonic stem cells. *Nat. Commun.* 7:10660 doi: 10.1038/ncomms10660 (2016).

## Supplementary Material

Supplementary Information Supplementary Figures 1-8
