# Supplementary material for: A short G1 phase imposes constitutive replication stress and fork remodelling in mouse embryonic stem cells
Source: Nat Commun. 2016 Feb 15;7:10660. doi: 10.1038/ncomms10660 (PMC4756311; doi:10.1038/ncomms10660)
Supplement: Supplementary Information — Supplementary Figures 1-8 [file ncomms10660-s1.pdf]

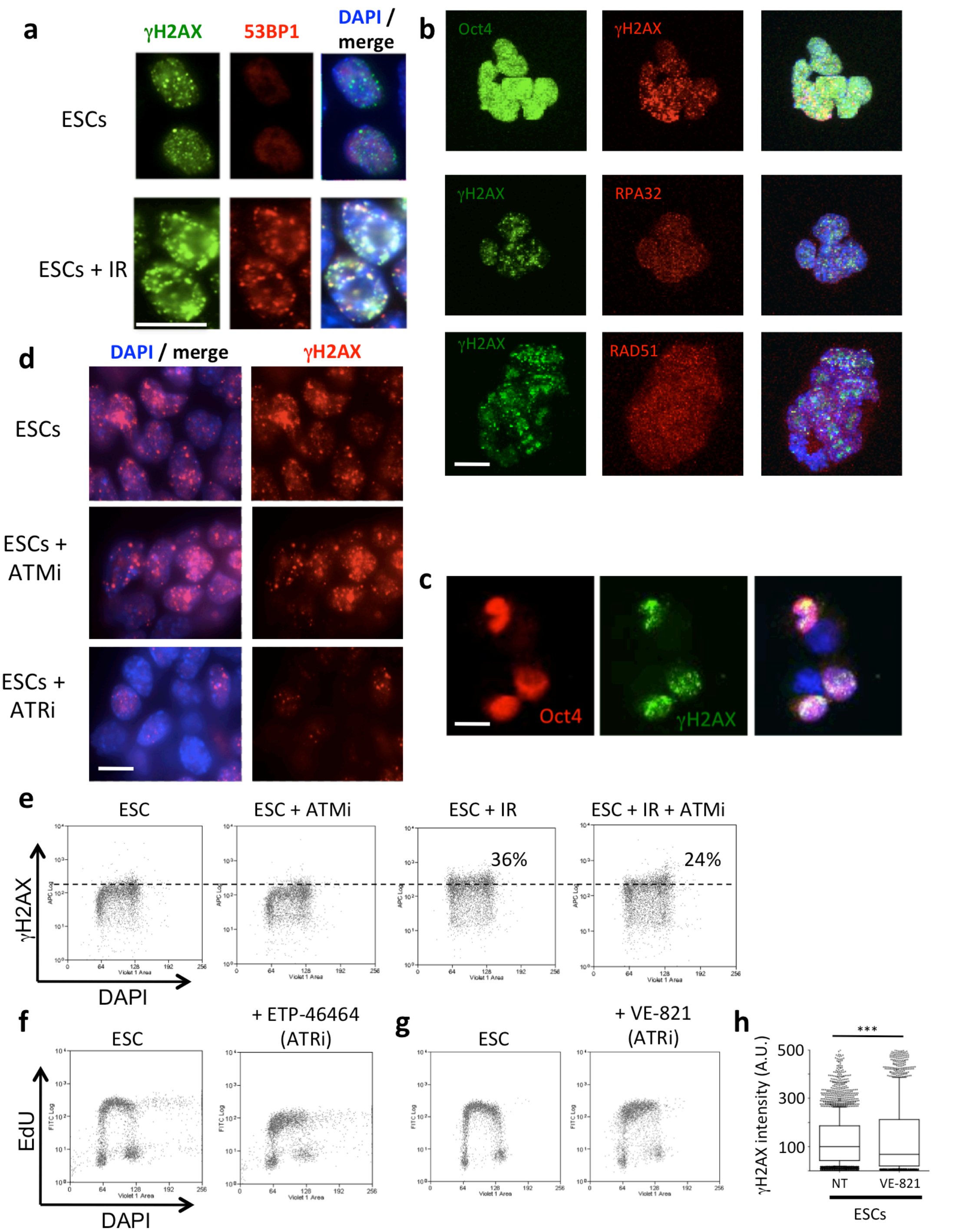

Supplementary Figure 1. γH2AX in ESCs coexists with chromatin loading of ssDNA binding proteins, is lost upon the induction of differentiation, is independent of cell line/cell culture conditions, and is ATR dependent. (**a**) Immuno-fluorescent (IF) staining of non-treated and irradiated (IR, 10 Gy) ESCs for the DNA damage marker γH2AX and the DSB specific marker 53BP1. (**b**) IF staining of ESC colony (*Stat3* cell line, derived and cultivated in 2i+LIF medium) for the stem cell marker Oct4, the DNA damage marker γH2AX and chromatin-bound ssDNA-binding proteins RPA32 and RAD51. (**c**) IF staining of ESCs (*JM8* cell line) upon partial differentiation (3d from LIF removal). (**d**) γH2AX IF staining in ESCs upon mock-, ATM inhibitor (ATMi, KU55933) or ATR inhibitor (ATRi, ETP-46464) treatment. Scale bars: 25 µm. (**e**) Control experiment to monitor activity of ATM inhibitor. Flow cytometric analysis of DDR activation (γH2AX) and DNA content (DAPI) in mock-treated ESCs and ESCs treated with the ATMi KU55933, optionally exposed to ionizing radiation (IR, 10 Gy). (**f, g**) Flow cytometric analysis of DNA synthesis (EdU incorporation) and DNA content (DAPI) in mock-treated ESCs and ESCs treated with the ATRi ETP-46464 (**f**) and VE-821 (**g**). (**h**) FACS-based quantitative assessment of γH2AX signal intensity in mock-treated ESCs and ESCs treated with the ATRi VE-821.

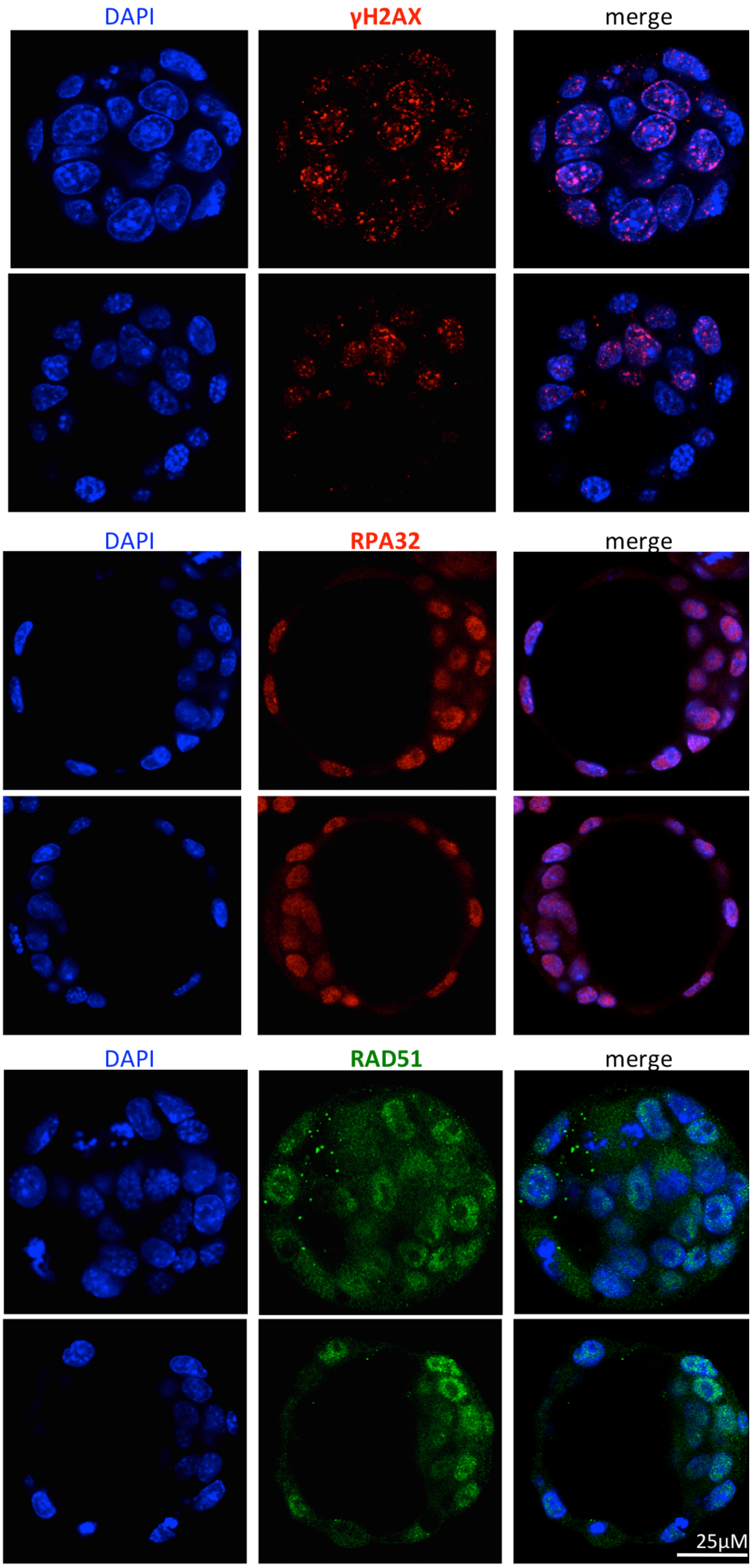

Supplementary Figure 2. Replication stress markers are detectable *in vivo*, in pre-implantation mouse embryos (blastocysts). Additional images of blastocysts isolated at day E3.5 and stained as in Fig. 1d for γH2AX, RPA32 and RAD51.

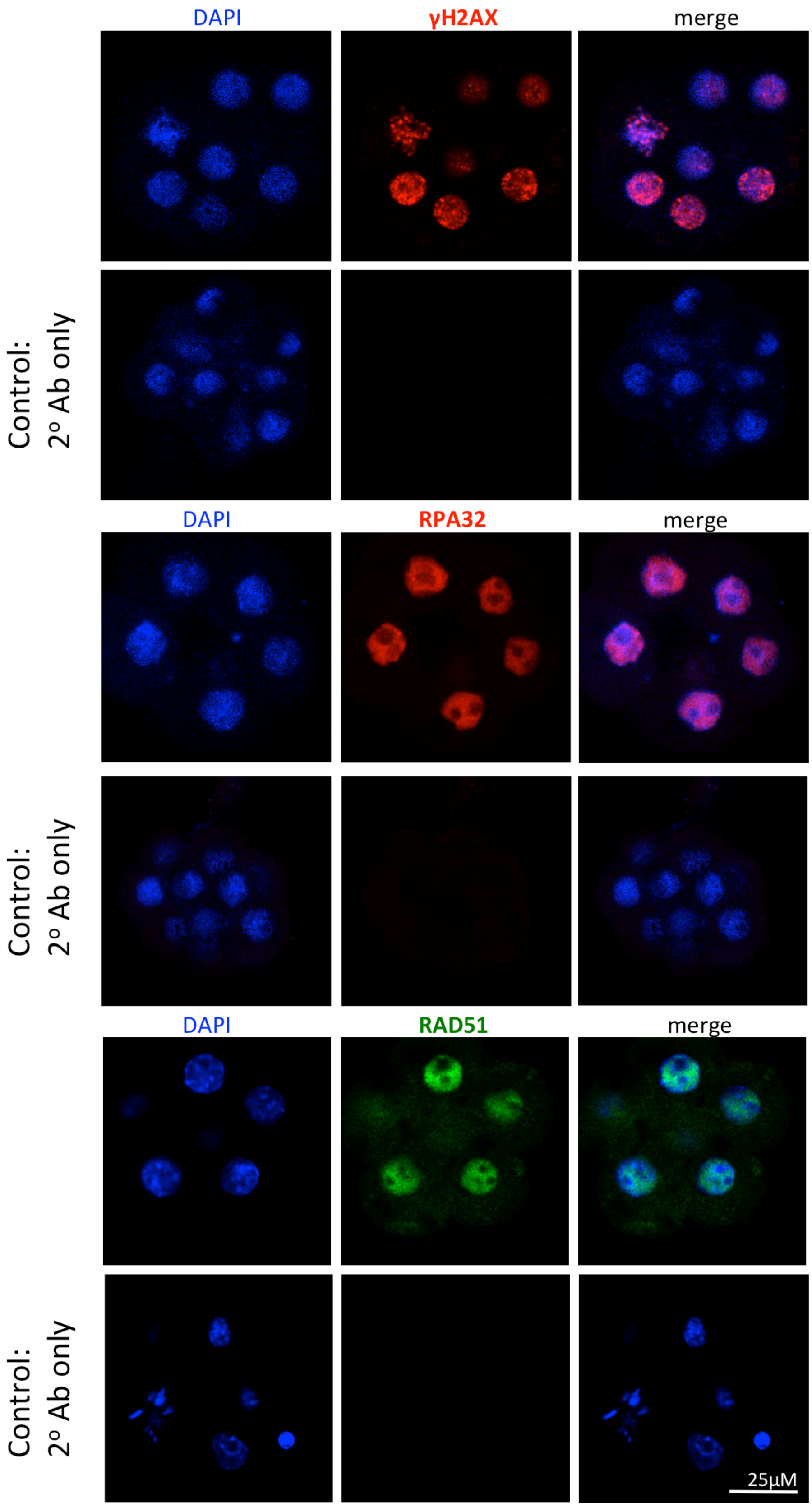

Supplementary Figure 3. Replication stress markers are detectable *in vivo*, in preimplantation mouse embryos (morulas). IF staining of morulas isolated at day E2.5 and stained for γH2AX, RPA32 and RAD51. Number of embryos analyzed per staining was 7, 6 and 4 respectively. Representative images are shown. IF stainings where the primary antibody was omitted are shown as negative controls.

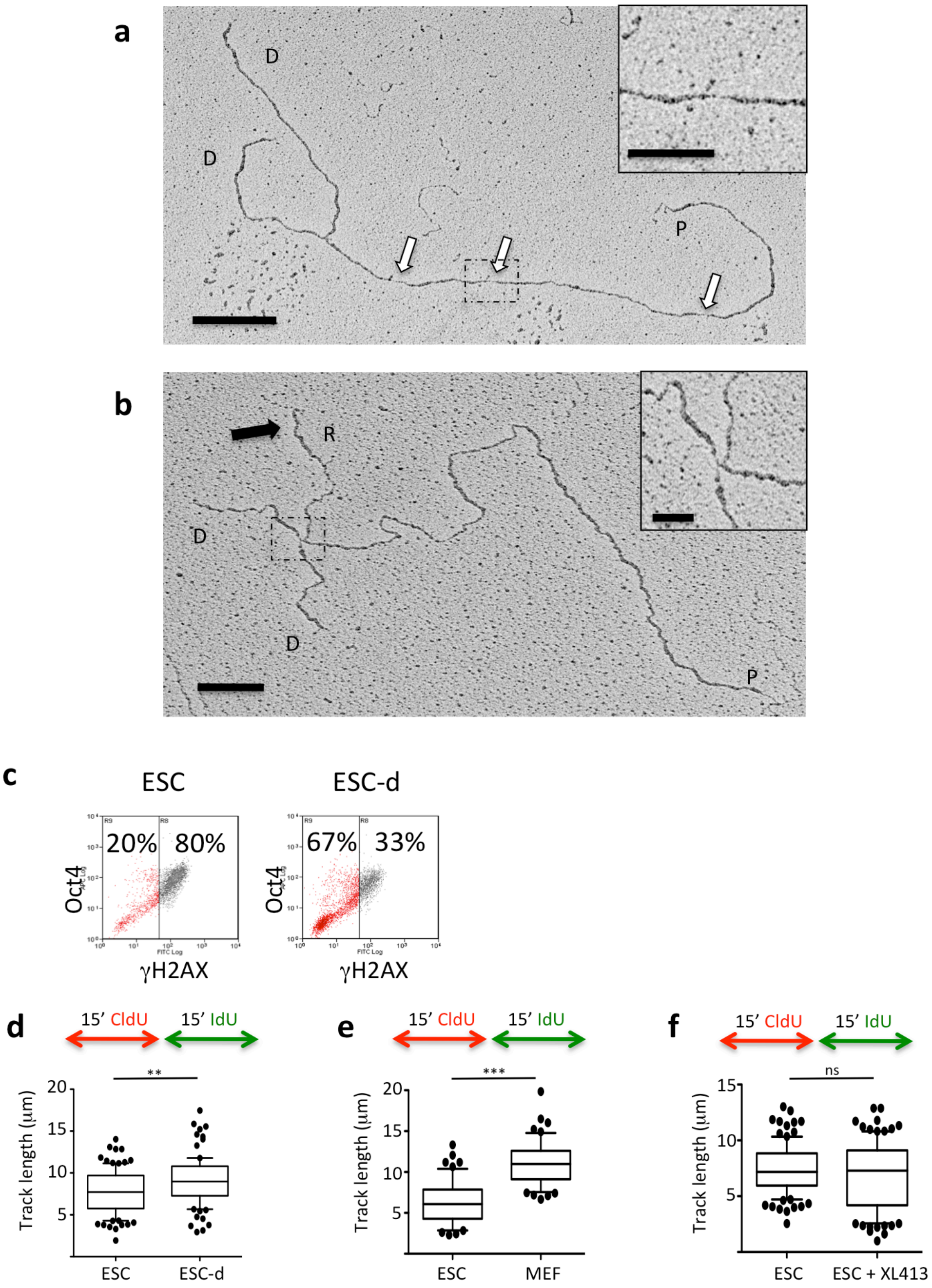

Supplementary Figure 4. ESCs display accumulation of ssDNA gaps and reversed forks. Replication fork slowing in ESCs is independent from origin density. (**a, b**) Additional electron micrographs of representative replication forks from ESCs (see also Fig. 2). The identification of parental (P) and daughter (D) duplexes, regressed arms (black arrow, R) and ssDNA gaps (white arrows), are based on length measurements and careful analysis of filament thickness and junctions (see Methods). Insets: in (**a**), a magnified ssDNA gap; in (**b**), the four-way junction at the reversed fork. Scale bars: 500bp (= 217nm), 200bp in inset. (**c**) FACS based quantification of differentiation. 80% of cells in an unperturbed ESC culture are positive for both Oct4 and γH2AX (top left panel). 5d after the induction of differentiation, 67% of the cells in the ESC-d sample are negative for both Oct4 and γH2AX (top right panel). Importantly, Oct4- γH2AX- cells (red subpopulation) at this stage of the differentiation procedure (5d) are still actively dividing and proliferating (see also Fig. 1b). (**d-f**) DNA fiber spreading on ESC and ESC-d using 2x15' labeling protocol. CldU/IdU-containing tracts were immunostained in red and green respectively. The graph shows the statistical analysis of IdU track length (Mann-Whitney test). XL413 is a potent and specific CDC7 inhibitor that prevents further origin firing during the labeling time. **, $p<0.005$;***, $p<0.0005$; ns, non significant.

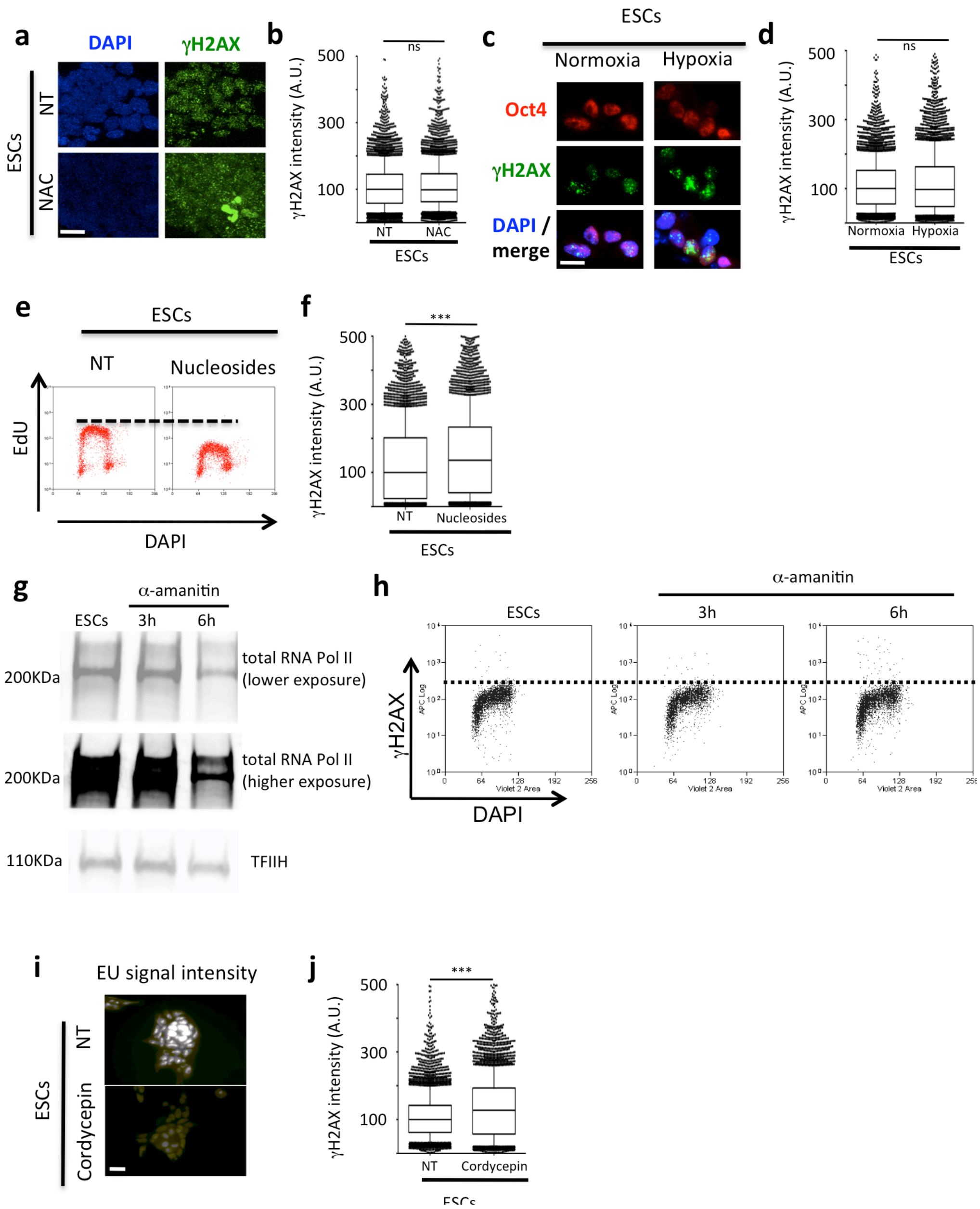

Supplementary Figure 5. Oxidative DNA damage, hypoxia, replication/transcription interference or nucleotide shortage are not the source of replication stress in ESCs. (**a**) IF stainings for γH2AX of mock-treated ESCs and ESCs treated with the potent reducing agent N-Acetylcysteine (NAC). No obvious difference in γH2AX levels is induced by the scavenging agent. (**b**) FACS-based quantitative assessment of γH2AX signal intensity in mock-treated and NAC-treated ESCs. No significant difference (ns) is observed. (**c**) Single cell IF staining for Oct4 and γH2AX on ESCs grown in normoxic (20% O_2) and hypoxic (1% O_2) conditions for 2 passages. (**d**) FACS based quantitative assessment of γH2AX signal intensity in ESCs grown in normoxic vs. hypoxic culture conditions. No significant difference (ns) is observed. (**e**) EdU incorporation in ESCs grown in minimal media (left panel) and ESCs grown in media containing an excess of nucleosides. The marked reduction in EdU incorporation proves that in these experimental conditions the nucleosides added in the media are effectively used during replication, outcompeting EdU. (**f**) FACS-based quantitative assessment of γH2AX signal intensity in ESCs grown in minimal media and ESCs cultivated in presence of excess nucleosides. Nucleoside addition causes a subtle increase in the detected levels of γH2AX in ESCs, as opposed to suppressing it. (**g**) Western blot analysis of total RNA pol II levels in mock-treated ESCs and ESCs treated with alpha-amanitin for 3h and 6h, respectively. TFIIH was used as loading control. (**h**) Flow cytometry analysis of γH2AX vs. DAPI incorporation in mock treated and alpha-amanitin treated ESCs. (**i**) EU incorporation visualized by IF in untreated and cordycepin-treated ESCs, showing that the treatment with cordycepin effectively blocks transcription (monitored by loss in EU incorporation). (**j**) FACS-based quantitative assessment of γH2AX signal intensity in untreated and cordycepin-treated ESCs. Treatment with cordycepin causes a subtle increase in the detected levels of γH2AX in ESCs, likely as a consequence of the genome-wide block of transcript elongation. Scale bars: 25 μm.

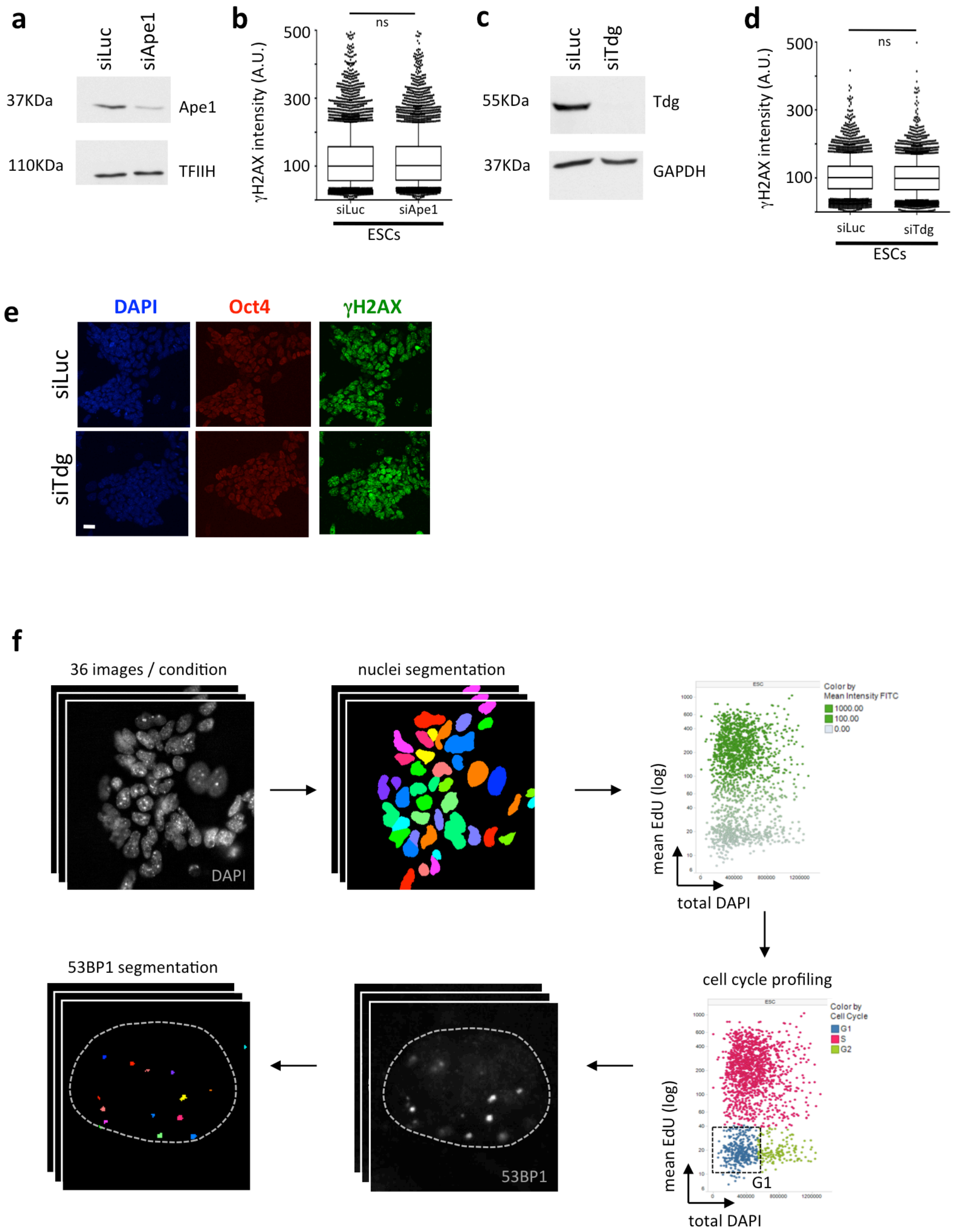

Supplementary Figure 6. Active DNA demethylation is not the source of replication stress in ESCs; QIBC-analysis of 53BP1 nuclear bodies and CDC7i arrest (**a**) Detection of Ape1 protein levels by Western blotting in control ESCs and upon siRNA-mediated Ape1-depletion. TFIIH is used as loading control. (**b**) FACS-based quantitative assessment of γH2AX signal intensity in mock transfected and Ape1-depleted ESCs. No significant difference (ns) is observed. (**c**) Detection of thymine DNA glycosylase (TDG) protein levels by Western blotting in control ESCs and upon siRNA-mediated TDG-depletion. GAPDH is used as loading control. (**d**) FACS-based quantitative assessment of γH2AX signal intensity in mock transfected and TDG-depleted ESCs. No significant difference (ns) is observed. (**e**) IF staining of mock transfected and TDG-depleted ESCs with Oct4 and γH2AX. Scale bar: 25 μm. No obvious difference in γH2AX levels is induced by effective TDG depletion. (**f**) Scheme for microscopy-based quantification of 53BP1 nuclear bodies in the G1 phase of ESCs. Asynchronous populations of ESCs were labelled with EdU and co-stained for 53BP1 and DAPI. Nuclei were segmented and cell cycle profiles were obtained based on EdU intensity and DNA content (DAPI). 53BP1 nuclear bodies were then quantified in cells with low EdU levels and low DNA content (see Methods for details).

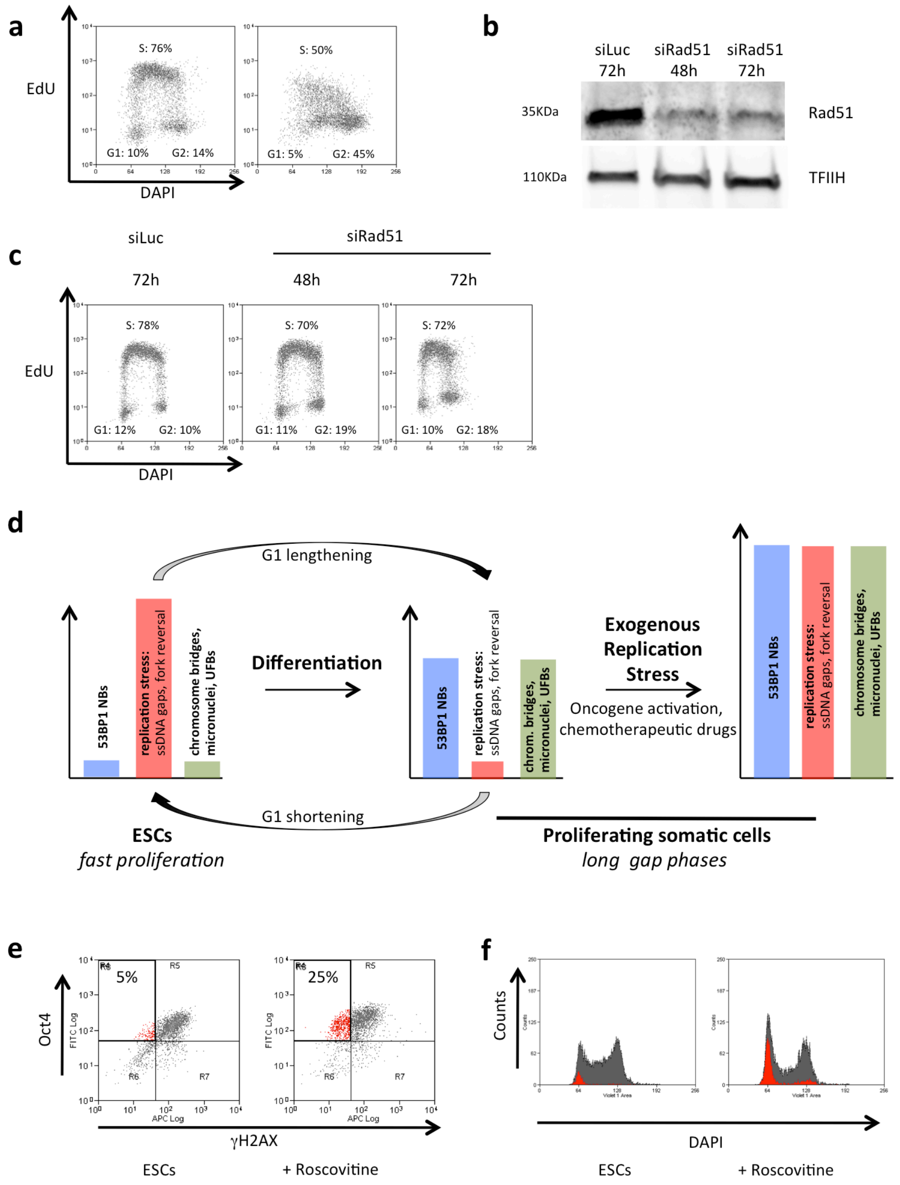

Supplementary Figure 7. PARP and Rad51 are essential for replication fork protection in ESCs; strategies for genome integrity in stem and somatic cells. (**a, c**) Flow cytometric analysis of DNA synthesis (EdU incorporation) and DNA content (DAPI) in mock-treated ESCs, ESCs treated with the PARP inhibitor Olaparib (**a**), mock-transfected ESCs and ESCs transfected with siRad51 for 48h and 72h (**c**). (**b**) Detection of Rad51 protein levels by Western blotting in control ESCs and upon siRNA-mediated Rad51-depletion for 48h and 72h. TFIIH is used as loading control. (**d**) Graphical summary of the different strategies for genome maintenance in ESCs and somatic cells. ESCs - that have fast proliferation as top priority during the few cell cycles they exist - have short gap phases, during which they tolerate high levels of unresolved replication stress, displaying minimal levels of 53BP1 nuclear bodies (NBs) in G1 and of unresolved mitotic structures in M. Consequently, they accumulate replication stress in S phase (ATR-dependent γH2AX), but effectively protect genome integrity by promoting fork reversal. Conversely, differentiated cells can spend more time in gap phases and attempt resolving their intrinsically lower levels of replication stress by assembling 53BP1 NBs in G1 and delaying mitosis onset to minimize unresolved mitotic structures. Experimentally induced G1 shortening in differentiated cells recapitulates the replication features of ESCs. Conversely, lengthening of the G1 phase in ESCs is sufficient to allow 53BP1 NB assembly and to reduce the observed levels of replication stress in S phase. When challenged by exogenous replication stress, somatic cells exploit at best all available strategies to protect genome integrity in each cell cycle phase, increasing 53BP1 NBs, reversed forks, and mitotic structures. (**e**) FACS analysis of Oct4 and γH2AX in non-treated ESCs and ESCs treated with Roscovitine. The top-left quadrant identifies the subpopulation of stem cells ($Oct4^{+}$) that display negative γH2AX staining. (**f**) Cell cycle distribution, assessed by FACS-based DNA content (DAPI), of the samples in (**e**). The total population is displayed in gray and the subpopulation of $Oct4^{+}$ $γH2AX^{-}$ in red.

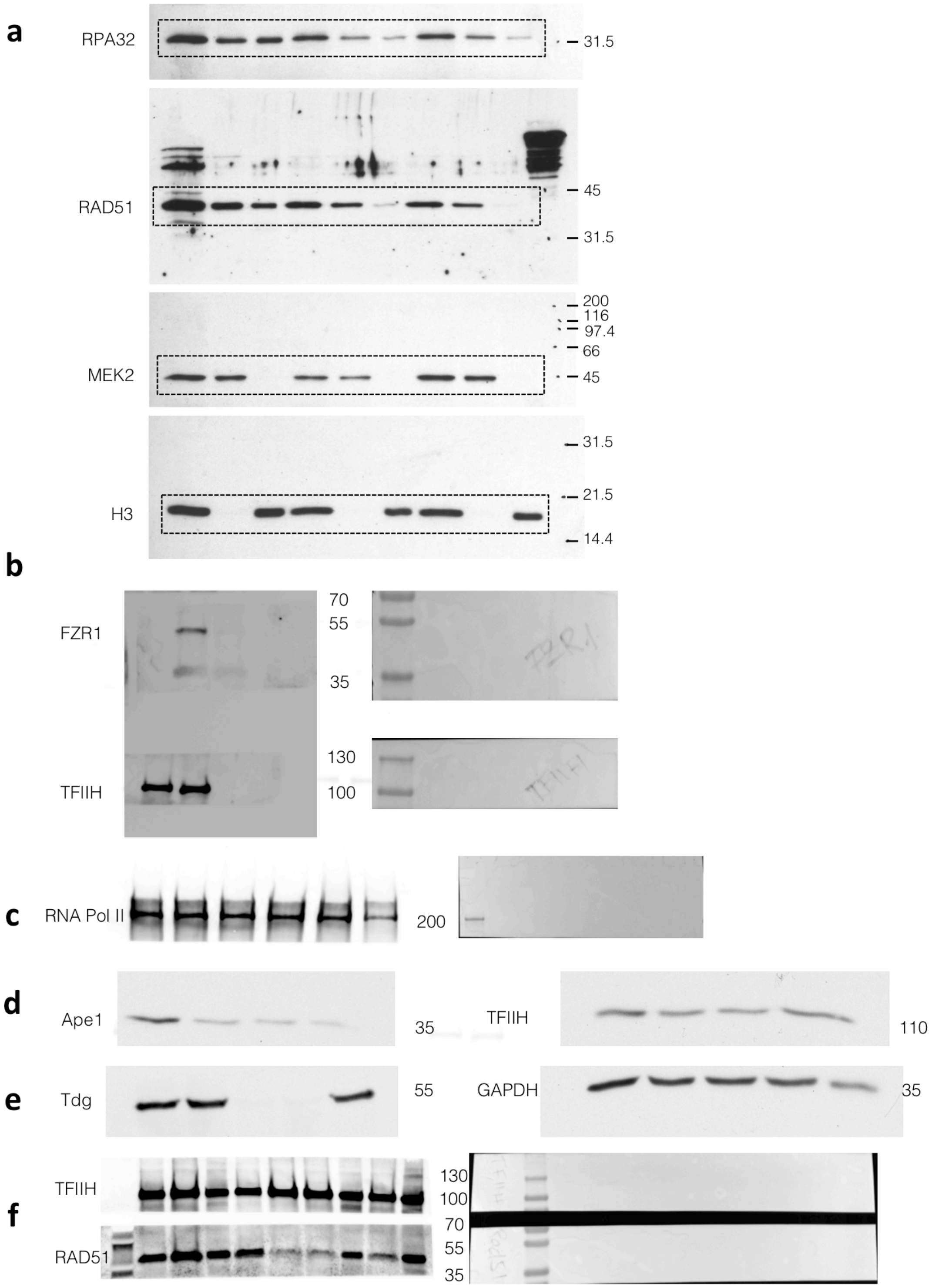

Supplementary Figure 8. Uncropped Westerns. a - f complete, uncropped blots with size markers corresponding to Westerns shown in Figures 1e (**a**), 4c (**b**), and Supplementary Figures 5g (**c**), 6a (**d**), 6c (**e**) and 7b (**f**).
